# Beveled vs. perpendicular skin incisions on the back: a case report

**DOI:** 10.3389/fsurg.2026.1951272

**Published:** 2026-10-05

**Authors:** Yong Dong, Huang Lin, Bo Li

**Affiliations:** Department of Aesthetic Plastic Surgery and Laser Medicine, Beijing Anzhen Hospital, Capital Medical University, Beijing, China

**Keywords:** back skin, beveled skin incision, hypertrophic scar, incision design, perpendicular skin incision, scar outcomes

## Abstract

This is a case report describing a within-patient self-controlled comparison of 45° beveled and 90° perpendicular skin incisions on the back in a 56-year-old female undergoing benign lipoma excision. Beveled skin incisions are routinely recommended in aesthetic surgery for their ability to enlarge dermal contact areas and facilitate favorable wound edge apposition, particularly in low-tension, thin cutaneous regions such as the face and neck. However, whether this cosmetic advantage extends to high-tension, thick-skin sites like the back remains unclear. This case report addresses this gap, and the specific novelty of this report is twofold: (1) it compares 45° beveled and 90° perpendicular incisions within the same patient and the same continuous dorsal wound, thereby reducing inter-individual confounding variables; (2) it focuses on a high-tension, thick-dermis, shear-prone dorsal site, whereas most prior evidence comes from low-tension facial or neck skin. This case report follows the CARE guidelines. One single continuous incision was split into two segments: the proximal segment adopted a 90° perpendicular full-thickness skin cut, and the distal segment a 45° beveled incision. All surgical steps and standardized postoperative anti-scar treatment were kept identical for both segments. Serial scar evaluations were conducted at 1, 3, 6, and 12 months postoperatively using the Vancouver Scar Scale (VSS) and the Patient and Observer Scar Assessment Scale (POSAS). At the 12-month follow-up, the beveled segment formed prominent, firm hypertrophic scars with pigment irregularities, while the perpendicular segment healed into flat, soft tissue with more favorable cosmetic outcomes. Differences in hypertrophic scar severity could be detected at every follow-up visit. However, exclusive causal attribution to incision angle cannot be confirmed, as subtle local differences between wound segments cannot be fully excluded. This case report is limited to a single patient and lacks comparison groups with intermediate bevel angles; therefore, it only offers hypothesis-generating preliminary observational evidence that the cosmetic advantages of beveled incisions may vary by anatomical site and may not translate to the back under high biomechanical stress. Observations raise the hypothesis that perpendicular skin incisions may merit consideration for dorsal operations; however, definitive clinical recommendations cannot be established from a single-patient experience alone.

## 1 Introduction

Surgical incision design is a critical modifiable determinant of postoperative scar quality and aesthetic outcomes in plastic surgery. Conventional principles recommend aligning incisions with relaxed skin tension lines and optimizing wound edge apposition to minimize scar formation (1, 2). Beveled incisions have gained wide acceptance because they increase dermal contact area, enrich fibroblasts and repair factors at the wound margin, and facilitate precise edge interlocking (3, 4). A beveled (oblique-angled) skin incision cuts the skin at an acute angle relative to the skin surface, in contrast to a perpendicular incision cutting at 90° to the epidermal plane; this technique is intended to enlarge dermal contact area and facilitate wound-edge eversion in low-tension regions such as the face (Figure 1). Multiple systematic reviews and comparative studies have demonstrated that 10°–45° beveled incisions yield superior long-term cosmetic outcomes compared with 90° perpendicular incisions (5–7), and they are now preferred for facelift and brow-lift procedures (8, 9).

**Figure 1:**
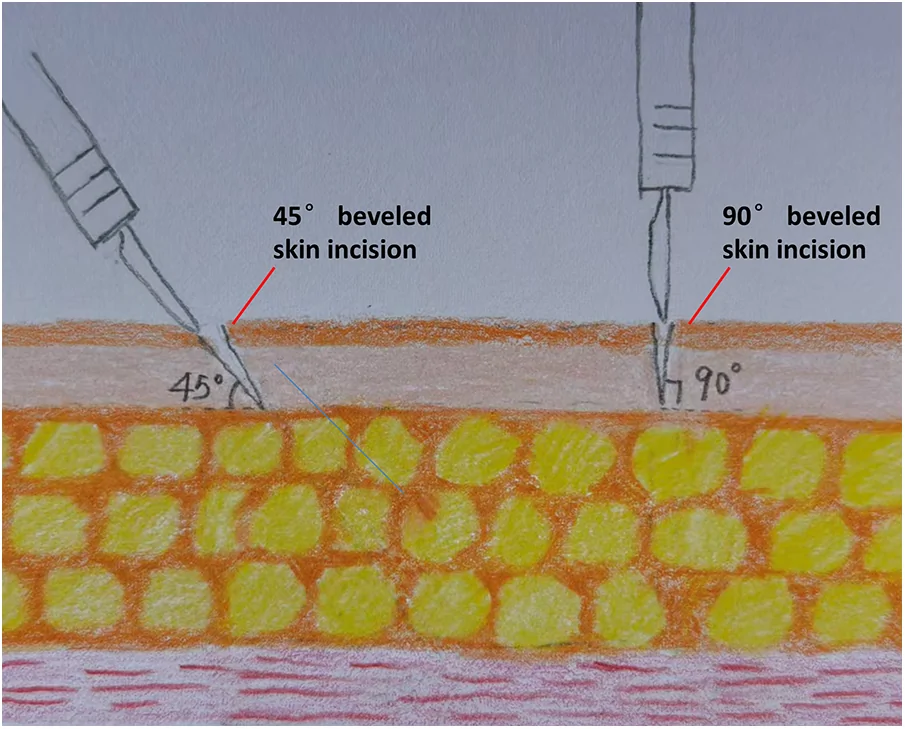
Schematic diagram comparing a 45° beveled skin incision and a 90° perpendicular skin incision.

However, nearly all existing evidence comes from thin, pliable skin areas with low static tension and minimal shear stress. The back, in contrast, possesses distinct anatomical and biomechanical properties: a thick, dense dermis with coarse collagen bundles, persistently high static tension, and repetitive dynamic shear forces from trunk and scapular movements (10). These characteristics create a high-load, high-micro-motion wound healing environment fundamentally different from that of the face or neck. To date, no controlled study has specifically addressed incision angle selection on the back, and no self-controlled trial within an identical dorsal surgical field has been reported. This has led to a generalized overestimation of beveled incisions and a lack of evidence-based guidance for dorsal incision planning.

This case report adheres strictly to the CARE guidelines and features a unique within-patient comparison of beveled versus perpendicular incisions along one continuous dorsal wound, reducing many inter-individual confounding variables that plague conventional comparative research. Scar assessments were performed by two board-certified attending plastic surgeons. The assessors were blinded to the incision-angle allocation of each wound segment. The validated original full-form POSAS version 2.0 was applied for all evaluations, incorporating both the Observer Scar Assessment Scale (OSAS) and Patient Scar Assessment Scale (PSAS) subscales. Each item was scored on a 10-point scale, where 1 represents normal-appearing skin and 10 represents the worst-imaginable scar. Clinical photographs were captured with a fixed digital camera, identical illumination, constant working distance, and consistent patient body positioning at each follow-up time-point. This study aimed to generate preliminary hypotheses regarding the optimal incision technique for dorsal cutaneous surgery and provide preliminary evidence for individualized incision design in high-tension anatomical sites.

## 2 Case presentation

A 56-year-old woman presented with a 20-year history of a painlessly, progressively enlarging subcutaneous mass in the left infrascapular region, and she had never received any medical evaluation previously. The complete clinical timeline is summarized in Table 1. Physical examination demonstrated a soft, freely mobile, non-tender subcutaneous lesion measuring approximately 9 cm in diameter (Figure 2A), with intact overlying skin without inflammatory or scar changes. Preoperative ultrasonography revealed a well-circumscribed, encapsulated mass with homogeneous hyperechoic signal, consistent with the diagnosis of lipoma.

**Table 1:** Timeline of clinical management for the patient.

Time point	Key clinical events
Initial visit	Clinical and imaging diagnosis of dorsal lipoma; informed consent obtained
Preoperative consultation	Physical examination+imaging confirmed benign dorsal lipoma; no history of keloid, diabetes or poor wound healing; full explanation of self-controlled incision design
Day of surgery	Routine skin disinfection, local infiltration anesthesia; single continuous incision divided into 90° perpendicular proximal segment and 45°beveled distal segment; complete lipoma resection, standardized three-layer layered suture; no intraoperative complications
Postoperative Day 7	Suture removal; both incision segments achieved primary healing, no hematoma/infection/dehiscence; uniform silicone gel+compression anti-scar treatment initiated
Postoperative Month 1	First follow-up; scar morphology palpation+VSS/POSAS quantitative scoring recorded
Postoperative Month 3	Second standardized scar assessment
Postoperative Month 6	Third standardized scar assessment
Postoperative Month 12	Final 12-month follow-up; long-term cosmetic outcome evaluation completed; patient subjective feedback collected

**Figure 2:**
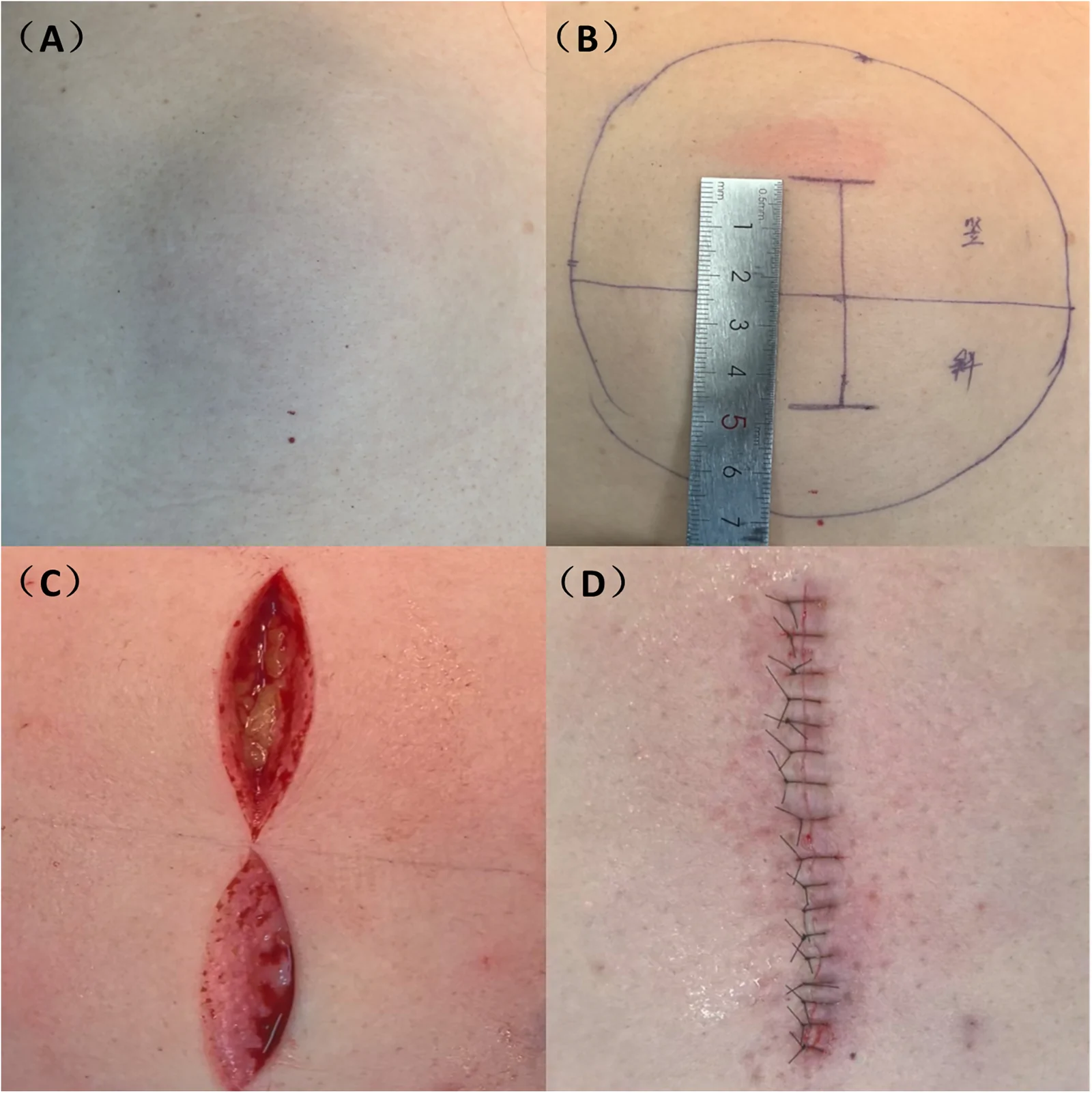
Perioperative images of dual-angle skin incision for large infrascapular lipoma excision. **(A)** Preoperative view of the subcutaneous lipoma mass. **(B)** Preoperative marking of the designed linear incision. **(C)** Intraoperative comparison of the cut edges of the 90° perpendicular proximal segment and 45° beveled distal segment. **(D)** Appearance of the wound after complete three-layer closure.

This intra-incision self-control design was adopted to compare long-term cosmetic scar outcomes between two cutaneous incision techniques. A single continuous linear incision was equally divided into proximal and distal segments. The proximal segment utilized a perpendicular 90° full-thickness cutaneous incision, while the distal segment was made with a standard 45° tangential beveled cut. After en-bloc resection of the lipoma, standardized three-layer layered closure was performed identically for both segments by a single senior plastic surgeon to avoid operator bias (Figures 2B–D). No intraoperative complications including hematoma, cutaneous ischemia or surgical site infection occurred, and both segments achieved uncomplicated primary healing. Histopathological examination confirmed benign lipoma.

Postoperatively, skin sutures were removed at week 1, followed by a unified 12-month regimen of topical silicone gel and compression scar therapy administered to both incision segments, with no additional local adjuvant treatments applied throughout follow-up.

Written informed consent was acquired from the patient for surgical intervention and subsequent publication of this case report. All procedures complied with the principles of the Declaration of Helsinki.

## 3 Diagnostic assessment, surgical intervention, and follow-up outcomes

### 3.1 Diagnostic assessment

Preoperative physical examination and ultrasonography confirmed a well-encapsulated, homogeneously hyperechoic subcutaneous mass without internal vascularity or invasive margins, consistent with a benign lipoma and effectively excluding malignant features or active inflammation.

### 3.2 Surgical intervention

En-bloc tumor excision was performed under local anesthesia. To isolate the effect of the incision technique, a single linear incision was equally divided into proximal (90° perpendicular full-thickness) and distal (45° beveled) segments, with both halves receiving identical three-layer closure, uniform suture spacing, and standardized hemostasis. Incision angle represented the main intended experimental variable; nonetheless, potential minor local anatomical and biological differences between the proximal and distal wound segments cannot be completely ruled out in this observational single-case report.

### 3.3 Follow-up and outcomes

Both incisions healed primarily without complications. Standardized scar assessments (VSS, OSAS, and PSAS) were conducted at 1, 3, 6, and 12 months postoperatively, with longitudinal data presented in Table 2. Representative clinical photographs were obtained at each time point to document visual morphological changes (Figure 3).

**Table 2:** Dynamic VSS and POSAS scar scores across four postoperative follow-up time points.

Follow-up Time	Scale & Items	45° Beveled Incision Segment	90° perpendicular Incision Segment
1 Month Postoperatively	Vancouver Scar Scale (VSS) Total Score	5	4
	Observer Scar Assessment Scale (OSAS) Total Score	29	21
	Patient Scar Assessment Scale (PSAS) Total Score	12	9
3Month Postoperatively	Vancouver Scar Scale (VSS) Total Score	6	5
	Observer Scar Assessment Scale (OSAS) Total Score	25	18
	Patient Scar Assessment Scale (PSAS) Total Score	12	9
6 Month Postoperatively	Vancouver Scar Scale (VSS) Total Score	6	5
	Observer Scar Assessment Scale (OSAS) Total Score	24	15
	Patient Scar Assessment Scale (PSAS) Total Score	12	8
12 Month Postoperatively	Vancouver Scar Scale (VSS) Total Score	8	4
	Observer Scar Assessment Scale (OSAS) Total Score	28	11
	Patient Scar Assessment Scale (PSAS) Total Score	14	8

**Figure 3:**
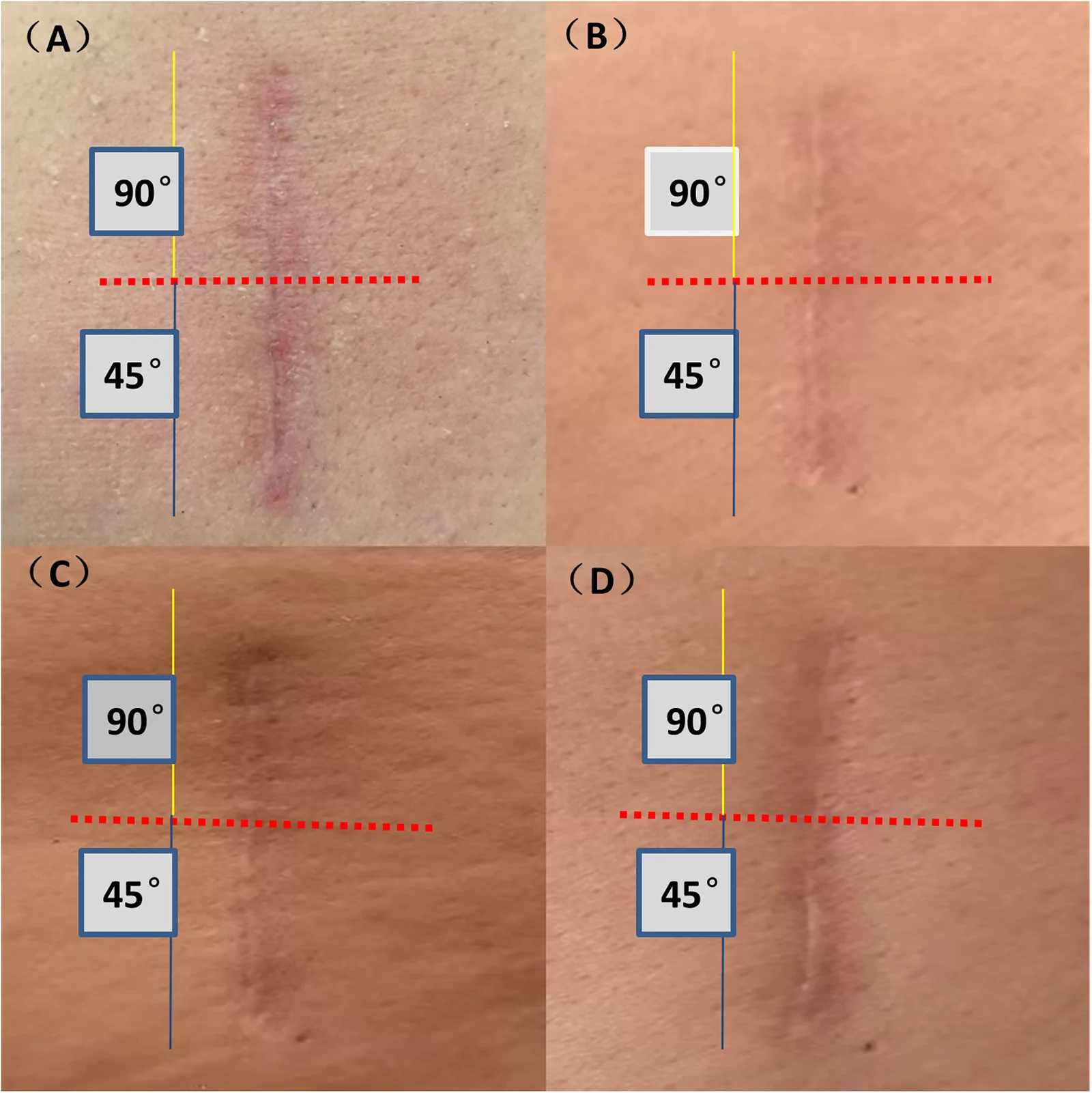
Postoperative scar morphology at sequential follow-up stages. **(A)** 1 month postoperatively; **(B)** 3 months postoperatively; **(C)** 6 months postoperatively; **(D)** 12 months postoperatively.

Throughout the 12-month follow-up, the perpendicular incision consistently demonstrated superior remodeling dynamics compared to the beveled counterpart. While the beveled segment exhibited persistent hypertrophic elevation, progressive firmness, and marked pigmentary irregularities, the perpendicular segment showed gradual flattening, softening, and near-complete pigment resolution over time. These divergent trajectories culminated at the 12-month endpoint, where the perpendicular segment achieved a supple, near-normotrophic scar, whereas the beveled segment developed a pronounced raised and dyspigmented scar (Table 2).

## 4 Discussion

### 4.1 Strengths and innovations of this self-controlled design

This case report is fully compliant with the CARE guidelines and adopts a within-patient observational comparison. The primary novelty of the present work lies in its within-incision, same-patient comparison of 45° beveled versus 90° perpendicular incisions within the dorsal high-tension skin environment; to our knowledge, no prior published study has adopted this exact study setup for back cutaneous surgery. This study substantially reduces many inter-individual confounding factors which cannot be ruled out in conventional cross-patient comparative studies; however, it does not fully eliminate all possible confounders, including subtle local differences between proximal and distal wound regions. Combined with standardized quantitative scar scales and serial clinical photographs, this research yields hypothesis-generating comparative observational outcomes. Most existing trials comparing different incision angles enroll separate patient cohorts and only focus on low-tension facial skin, inevitably introducing individual bias (3, 5). To the best of our knowledge, this is the first self-controlled comparison of beveled and perpendicular incisions on identical dorsal tissue, filling an unaddressed research gap and providing preliminary observational evidence that raises the hypothesis that the cosmetic efficacy of incision angles varies by anatomical biomechanical properties.

### 4.2 Mechanisms underlying severe hypertrophic scarring after dorsal beveled incisions

Few published papers systematically compare wound healing differences between beveled and perpendicular cuts. Most prior clinical reports hold that beveled incisions produce superior cosmetic effects by expanding dermal contact areas and reducing infection risk (5, 11, 12), while a minority of histological studies detect no long-term healing differences between the two incision modes (13). Our clinical findings differ from mainstream aesthetic surgery consensus, and three non-validated, plausible mechanistic hypotheses may potentially explain this discrepancy, although none were directly tested in the present case. First, oblique cutting may induce excessive tissue trauma and prolonged inflammatory response. The dorsal dermis is thick and packed with dense collagen bundles. A 45° beveled cut might transect more layers of dermal fibers, potentially leading to extensive hidden soft tissue injury, sustained inflammatory activation and excessive fibroblast proliferation. Such severe tissue damage rarely occurs when beveled incisions are applied to thin facial skin. Second, persistent shear stress could trigger recurrent wound micromotion. Static tension alone cannot drive pathological scar formation; sustained transverse shear force and micro-slip along wound margins are key triggers for abnormal collagen deposition (10, 14). Dorsal skin bears constant shear stress during torso and scapular movement. Perpendicular incisions may maintain symmetric, stable wound edge opposition against friction, whereas asymmetric beveled wound margins repeatedly shift, disrupting normal tissue remodeling balance. Third, beveled incisions show poor adaptability to thick, stiff dorsal skin. The skin eversion advantage of oblique cuts only applies to thin, pliable facial dermis. Thick, rigid back skin may tend to curl inward after beveled cutting, potentially forming tiny subcutaneous dead spaces and continuous inflammatory infiltration that delays wound maturation and aggravates scar hyperplasia.

### 4.3 Clinical implications and study limitations

We caution against directly translating incision schemes optimized for low-tension facial skin to the back; however, definitive clinical practice recommendations cannot be derived solely from this single-patient observation. For dorsal lipoma resection and other cutaneous operations, the present case generates a hypothesis that 90° perpendicular full-thickness incisions may minimize collagen damage and wound micro-movement, potentially lowering the risk of hypertrophic scarring and delivering better long-term cosmetic results. Beveled incisions are well-suited for low-tension, thin-dermis sites including face, neck and scalp, where they can enhance wound-edge eversion and dermal apposition. However, these cosmetic advantages may not straightforwardly extrapolate to high-tension sites with thick dermis and recurrent shear forces such as the back, posterior chest, and scapular region. We propose the hypothesis that anatomical-site-specific biomechanical properties should guide selection of incision-angle technique rather than universal adoption of beveled cutting across all body regions; formal validation in larger studies is required.

Langer's lines originate from static cadaveric measurements and advise incisions oriented parallel to skin tension lines to reduce hypertrophic scar risk. Nevertheless, the living dorsal skin undergoes dynamic shear stress during trunk motion that cannot be predicted by cadaver-based maps. In our case, tumor boundaries forced a longitudinal incision that deviated from transverse Langer's lines in the infrascapular area, representing a directional disadvantage shared by both incision segments. Even under this suboptimal orientation, the 45° beveled segment developed worse hypertrophic scars at every follow-up visit compared with its perpendicular counterpart. This single-case observation generates a hypothesis that incision angle may act as an independent-acting factor influencing dorsal scar performance, separate from incision direction relative to tension lines; definitive inference cannot be drawn from one single case. From a mechanistic perspective, it is hypothesized that beveled cutting disrupts more dermal collagen bundles and creates asymmetric wound edges vulnerable to shear-related micromotion, impairing collagen maturation. Perpendicular incisions are hypothesized to minimize dermal injury and maintain stable wound-edge apposition. Based on this single-patient observation, when tension-line-parallel incisions are not feasible on the back due to anatomical constraints, clinicians might consider perpendicular full-thickness cuts over beveled incisions as one plausible option, rather than receiving this as a firm clinical guideline.

Incision orientation (horizontal versus vertical relative to bod*y* axis) also interacts with scar formation on the back. Even with identical incision angle (beveled or perpendicular), the wound direction relative to local skin tension determines baseline wound-edge shear and traction. In our present case, the entire continuous wound adopted a single longitudinal orientation; we cannot draw conclusions regarding horizontal-oriented back incisions. Horizontal back incisions may experience different vector forces from scapular and trunk movement. Further studies should separately evaluate the interaction between incision orientation (horizontal/vertical) and incision angle (beveled/perpendicular) for dorsal cutaneous surgery.

Several inherent limitations exist in this single-case observation: only one patient was enrolled; control groups with 10°−30° mild beveled incisions were not set; the 12-month follow-up period fails to capture fully mature scar morphology. The dorsal infrascapular region is covered by routine clothing in most cultural contexts. For many patients, cosmetic visibility of back scars may be of relatively low priority compared with functional outcomes. Therefore, the clinical impact of our observed scar differences may vary according to individual patient expectations and cultural background. Although VSS and POSAS measurements provide objective scar assessment in this single-patient self-controlled observation, large-sample, multi-center prospective case series with longer-term follow-up beyond 12 months are urgently required to validate our preliminary findings, explore intermediate bevel angles, and establish evidence-based incision-angle recommendations for high-tension body regions.

## 5 Patient perspective

One year after surgery, the patient stated an obvious cosmetic disparity between the two incision zones. The 45° beveled scar remained raised, hard and unsightly, and it produced mild pruritus during torso movement; the perpendicular scar was flat and barely visible under daily clothing. The patient indicated a preference for perpendicular incisions if future back surgery is required, and fully agreed to de-identified publication of all clinical data and subjective feedback. Separate written informed consent for recording and publishing personal experience was obtained from the patient.

These subjective observations reflect only this individual patient's experience and should not be extrapolated to broader patient populations.

## Data Availability

The original contributions presented in the study are included in the article/Supplementary Material, further inquiries can be directed to the corresponding author.
